# *Ex Vivo* Neurogenesis within Enteric Ganglia Occurs in a PTEN Dependent Manner

**DOI:** 10.1371/journal.pone.0059452

**Published:** 2013-03-19

**Authors:** Laren Becker, Johann Peterson, Subhash Kulkarni, Pankaj Jay Pasricha

**Affiliations:** 1 Division of Gastroenterology and Hepatology, Department of Medicine, Stanford University, Stanford, California, United States of America; 2 Department of Pediatrics, University of California Davis, Sacramento, California, United States of America; 3 Division of Gastroenterology and Hepatology, Department of Medicine, Johns Hopkins University, Baltimore, Maryland, United States of America; Temple University School of Medicine, United States of America

## Abstract

A population of multipotent stem cells capable of differentiating into neurons and glia has been isolated from adult intestine in humans and rodents. While these cells may provide a pool of stem cells for neurogenesis in the enteric nervous system (ENS), such a function has been difficult to demonstrate *in vivo*. An extensive study by Joseph et al. involving 108 rats and 51 mice submitted to various insults demonstrated neuronal uptake of thymidine analog BrdU in only 1 rat. Here we introduce a novel approach to study neurogenesis in the ENS using an *ex vivo* organotypic tissue culturing system. Culturing longitudinal muscle and myenteric plexus tissue, we show that the enteric nervous system has tremendous replicative capacity with the majority of neural crest cells demonstrating EdU uptake by 48 hours. EdU^+^ cells express both neuronal and glial markers. Proliferation appears dependent on the PTEN/PI3K/Akt pathway with decreased PTEN mRNA expression and increased PTEN phosphorylation (inactivation) corresponding to increased Akt activity and proliferation. Inhibition of PTEN with bpV(phen) augments proliferation while LY294002, a PI3K inhibitor, blocks it. These data suggest that the ENS is capable of neurogenesis in a PTEN dependent manner.

## Introduction

The recognition that neurogenesis occurs in defined regions of the adult central nervous system (CNS) via a population of neural stem cells (NSCs) [1], [2], [3], [4] has provoked speculation that a similar phenomenon occurs in the enteric nervous system (ENS) [5]. The ENS is a complex network of neural crest-derived neurons and glia that span the gastrointestinal tract and control complex gut behaviors including peristalsis, secretion, and blood flow. Given their location in the gastrointestinal tract, enteric neurons face a variety of insults including the mechanical stress of gut motility, toxins absorbed from the bowel, and inflammatory/infectious processes from enteric pathogens. Thus, it seems logical to expect neurogenesis in the ENS. Indeed, so-called enteric neural stem cells (ENSCs) have been isolated from embryonic and adult intestine using a variety of techniques including growth selection and selective sorting either by cell surface markers for Ret, p75, and CD49b (alpha_2_-integrin) or fluorescent proteins [6], [7], [8], [9], [10], [11], [12], [13], [14], [15], [16], [17], [18], [19], [20], [21]. In culture, these cells demonstrate properties of stemness including self-renewal and multipotency (differentiating into neurons, glia and myofibroblasts) [8], [12]. They are also capable of colonizing gut tissue *ex vivo* and imparting functional changes to the neuromuscular activity of these explants [14].

However, despite the propensity of ENSCs for neurogenesis *in vitro*, a similar function has proven challenging to demonstrate *in vivo* [11]. Several ENS injury models have demonstrated significant neural plasticity, which suggests neurogenesis, but these studies failed to differentiate between new neurons and existing neurons that migrated from bordering regions [22], [23], [24], [25]. Studies using the thymidine analog bromodeoxyuridine (BrdU) to label dividing cells have had variable success demonstrating neurogenesis *in vivo* [11], [15], [26]. While BrdU uptake was not observed in neurons under steady-state conditions in post-natal mice, it was seen following treatment with a 5-hydroxytryptamine receptor 4 (5-HT_4_) agonist [15]. However, Joseph et al. performed an exhaustive study involving 108 rats and 51 mice under a variety of conditions including steady-state, aging, pregnancy, diabetes, dietary modifications, exercise, inflammation, and injury, yet only saw BrdU uptake in a single rat following benzalkonium chloride (BAC) treatment [11]. Some groups have utilized genetic fate mapping with loxp/cre recombinase systems under an inducible promoter to evaluate for neurogenesis *in vivo* [11], [13]. Lineage tracing with *Sox10-creERT2* mice failed to show neurogenesis under steady-state conditions (after post-natal day 84). However, using an ENS injury model with BAC, a neurotoxic detergent, 9% of neurons surrounding the site of injury were of Sox10-origin, suggesting neurogenesis [13]. A similar lineage tracing system using *GFAP-creERT2* mice failed to show appreciable GFAP-derived neurons following BAC treatment, however this difference may be explained by the low efficiency of cre recombination (3%–5%) in these mice [11].

In light of the apparent discrepancy regarding the neurogenic potential of ENSCs *in vitro* and *in vivo*, there is a need to better understand the pathways that control this process. Phosphatase and tensin homolog deleted on chromosome ten (PTEN) is a lipid and protein phosphatase that has a key regulatory role in cell proliferation and survival by suppressing phosphatidylinositol-3-OH kinase (PI3K)/Akt signaling [27], [28]. PTEN appears to maintain stem cells in a quiescent state, with PTEN deletions causing increased proliferation in neural stem cells, haematopoietic stem cells and embryonic stem cells [29], [30], [31], [32], [33], [34], [35]. Tissue specific deletion of PTEN in the ENS resulted in hyperplasia of enteric ganglia [36], suggesting that PTEN may have a similar role in ENSCs. Using mice in which Cre recombinase under the Wnt1 neural crest specific promoter drives tdTomato expression in cells of neural crest origin, we employed an *ex vivo* organotypic tissue culture system with the thymidine analog ethynyldeoxyuridine (EdU) to study proliferation and neurogenesis in the ENS. After an initial lag phase, we find abundant EdU uptake within neurons and glia in the myenteric plexus. We find that the switch from quiescence to proliferation corresponds to a decrease in PTEN mRNA expression and an increase in phospho-PTEN (inactive form [37]). We also observe that inhibiting PTEN with potassium bisperoxo(1,10-phenanthroline)oxovanadate [bpV(phen)], augments proliferation in cells of neural crest origin.

## Materials and Methods

### Ethics Statement

Experimental protocols were approved by the Administrative Panel on Laboratory Animal Care (*APLAC*) at the Stanford University, California in accordance with the guidelines provided by the National Institutes of Health.

### Animals

A *Wnt1-cre:tdTomato* line was created by crossing B6.Cg-Tg(*Wnt1-cre*) (Jackson Laboratories) with *loxP-stop-tdTomato* (kindly provided by B. Barres) [7]. *Wnt1-cre;tdTomato* compound heterozygote mice between 2–3 months of age were used in the experiments.

### Tissue Preparation and *ex vivo* Organotypic Culturing

Mice were anesthetized with isofluorane and euthanized by cervical dislocation. A laparotomy was performed and the small intestine was removed and lavaged with PBS containing penicillin-streptomycin (PS; Invitrogen, Carlsbad CA). Small intestine was cut into segments of 2 cm in length and placed over a sterile plastic rod. A superficial longitudinal incision was made along the serosal surface and the longitudinal muscle with the adherent myenteric plexus (LMMP) was peeled off from the underlying tissue using a wet sterile cotton swab and placed in Opti-MEM containing PS. LMMP was cultured in stem cell medium (SCM) consisting of Neurobasal medium containing B27, 2 mM L-glutamine and 100 U/ml PS. EdU (Invitrogen, Carlsbad CA) was added to media at a final concentration of 25 µM. BpV(phen) (BioVision, Milpitas CA) was added to culture media at a final concentration of 200 or 400 nM, and LY294002 (Cell Signaling, Danvers MA) was added at a final concentration of 10 or 50 µM. The following growth factors were added to SCM for all experiments except those involving inhibitors or differentiation: fibroblast growth factor (bFGF, 10 ng/ml), epidermal growth factor (EGF, 10 ng/ml) and glial cell-derived neurotrophic factor (GDNF, 10 ng/ml) (Invitrogen, Carlsbad CA). Tissue was cultured at 37°C and 5% CO_2_, and medium was replaced every day. For differentiation experiments, LMMP was cultured in SCM containing EdU for 48 h before chasing in differentiating media (SCM without growth factors bFGF, EGF and GDNF) for an additional 5 days. For flow cytometry and microwell experiment, LMMP was digested in Digestion Buffer consisting of M199 media (Invitrogen, Carlsbad CA) containing 0.1% BSA, 1 mM CaCl_2_, 20 mM HEPES, 150 µM P188, 50 U/ml DNAse I (Worthington, Lakewood NJ), 1.1 mg/ml collagenase (Sigma, St. Louis MO), and dispase 1 mg/ml (Sigma, St. Louis MO) for 40 minutes at 37°C and 5% CO_2_. Tissue was washed in PBS with 1% BSA then passed through a 40 µm nylon mesh cell strainer before fixation.

### EdU Uptake and Immunofluorescence

Tissue was fixed in 4% paraformaldehyde (PFA) in PBS for 30 minutes at room temperature and permeabilized in 50% glycerol in PBS for 1 hour at room temperature. EdU imaging was performed with Alexa Fluor 488 azide using Click-iT EdU Imaging Kits (Invitrogen, Carlsbad CA) according to the manufacturer’s instructions. Following EdU labeling, whole mounts were blocked in PBS containing 10% normal goat serum (NGS) for 1 hour at room temperature before incubating at 4°C with primary antibodies diluted in PBS containing 1.5% NGS and 0.01% sodium azide for 3 days. The following antibodies were used: GFAP (rabbit 1∶1500, DAKO, Carpinteria CA), S100 (rabbit 1∶250, DAKO, Carpinteria CA), PGP9.5 (rabbit 1∶500, DAKO, Carpinteria CA), nNOS (rabbit 1∶250, Invitrogen, Carlsbad CA), βIII-tubulin (Tuj) (rabbit 1∶200, Sigma, St. Louis MO), and VIP (rabbit 1∶500, Abcam, Cambridge MA). After washing with PBS, tissue was incubated overnight at 4°C with secondary antibodies. The following secondary antibodies were used: Dylight 647 Anti-Rabbit (1∶300, Jackson Immuno, West Grove PA) and Brilliant Violet 421 anti-rabbit (1∶100, BioLegend, San Diego CA). After washing with PBS, coverslips were mounted onto glass slides using mounting media with or without DAPI (Vector Labs, Burlingame CA). Samples were examined and imaged with a CCD cooled camera on a Nikon C1 confocal microscope.

### Flow Cytometry

EdU labeling was performed with Alexa Fluor 488 azide using Click-iT EdU Flow Cytometry Assay Kit (Invitrogen, Carlsbad CA) according to the manufacturer’s instructions. Following blocking with 10% NGS, cells were incubated with primary antibodies for 20 min at 4°C. Antibodies to cell surface antigens were incubated prior to permeabilization while those to intracellular antigens were incubated after saponin-permeabilization. The following primary antibodies were used: Alexa Fluor 647 CD49b (1∶100, BioLegend, San Diego CA), p75 (rabbit 1∶100, Abcam, Cambridge MA), nestin (mouse 1∶100, Milipore, Billerica MA), GFAP (rabbit 1∶200, DAKO, Carpinteria CA), S100 (rabbit 1∶100, DAKO, Carpinteria CA), PGP9.5 (rabbit 1∶200, DAKO, Carpinteria CA), βIII-tubulin (rabbit 1∶100, Sigma, St. Louis MO), VIP (rabbit 1∶500, Abcam, Cambridge MA) and nNOS (rabbit 1∶100, Invitrogen, Carlsbad CA). Cells were pre-incubated with Mouse on Mouse blocking reagent (Vector Labs, Burlingame CA) for 30 minutes at room temperature when staining for the intracellular protein Nestin. Cells incubated without primary antibody were used for negative control. After washing, cells were incubated with secondary antibody when unconjugated primary antibodies were used. The following secondary antibodies were used: APC anti-rabbit and Dylight 647 anti-mouse (both 1∶100, Jackson Immuno, West Grove PA). Flow cytometry was performed on a LSR II (BD Bioscience, San Jose CA) and analyzed using FlowJo Software (Tree Star Inc, Ashland OR).

### Sphere Formation Assay

At the end of 48 h incubation in SCM, LMMP were pulsed with EdU for 12 h then dissociated in Digestion Buffer. Cells were washed in PBS with 10% FBS, passed through a sterile 40 µm nylon mesh cell strainer, and cultured in SCM for 3 days until neurosphere-like bodies (NLBs) were easily visualized. These primary NLBs were transferred to fibronectin-coated 60-mm plates (BD Biosciences) and cultured as an adherent monolayer. Cells were dissociated into single cells with Accutase (Invitrogen) and either layered on microwells (∼5000 cells/microwell grid) or cultured on fibronectin-coated plates for additional passage. Images of cells in microwells were captured on inverted fluorescence microscope at 4× objective. Cells were cultured in microwells for 6–8 days to allow sphere formation. Microwells were fixed, permeabilized and the Click-IT EdU reaction performed before imaging with a CCD cooled camera on a Nikon C1 confocal microscope at 60× objective. For sphere cell counts, cross-sectional images of NLBs were captured using confocal microscopy and spheres were counted for total number of cells (using DAPI) and number of EdU^+^ cells. Only those spheres that could be traced to a single cell at day 1 were included in analysis. The experiment was performed in biologic replicates using 3 animals and data was pooled for analysis.

### Microwells

Microwells for cell culture were formed by curing PDMS (Dow Corning Sylgard 184) on a mold to obtain a thin sheet of elastomer having an array of cubical wells 50 µm on each side. The elastomer sheets were numbered allowing individual wells to be located and photographed from day to day.

### Western Immunoblotting

Tissues were homogenized in Ripa Buffer (Sigma) supplemented with 1 mM phenylmethylsulfonyl fluoride, 0.5 mM NaF, Protease Inhibitor Cocktail (1∶100, Sigma), and Phosphatase Inhibitor Cocktail 2 (1∶100, Sigma). Lysates were clarified by centrifugation at 15,000 g for 20 min at 4°C. Following boiling in LDS sample buffer containing ß-mercaptoethanol (Invitrogen), 15 µg of protein (determined using Pierce BCA Protein Assay Kit, Rockford IL) was subjected to electrophoresis on 4–12% NuPAGE Bis Tris gels (150 V for 100 min, Invitrogen, Carlsbad CA) and transferred to 0.45 µm Hybond-P PVDF blotting membranes (110 V for 1 h, GE Healthcare, Pittsburgh PA). After washing and blocking in tris-buffered saline and 0.1% Tween 20 (TBST) containing 5% BSA for 1 h at room temperature, membranes were incubated with primary antibodies overnight at 4°C. The following primary antibodies were used: GAPDH (1∶2000, Abcam, Cambridge MA), PTEN, phospho-PTEN (Ser380), Akt, and Phospho-Akt (Ser473) (all 1∶1000, Cell Signaling, Danvers MA). Semi-quantative measurements of band intensity using pixel intensity were performed using ImageJ (NIH) and expressed in densitometric units normalized to the loading control (GAPDH).

### RNA Isolation and qRT-PCR

RNA from tissue was extracted using Qiagen RNeasy mini kit (Qiagen, Valencia CA), quantified using Nanodrop 2000c and converted to cDNA using High Capacity RNA-to-cDNA kit (ABI, Foster City CA). Quantification of PTEN expression was carried out using TaqMan Gene expression Assays and ABI StepOne plus real time instrument with the Taqman probes (ABI, Foster City CA) to PTEN (Mm00477208_m1) and HPRT (Mm01545399_m1). All experiments were performed in biological triplicates. Expression of each gene was normalized to the housekeeping gene HPRT. Fold change in gene expression between groups was calculated using the Pfaffl method.

### Statistical Analysis

Data is expressed as mean +/− standard error of mean or +/−95% confidence interval, as noted, and was analyzed using Student’s unpaired two-tailed *t*-test, ANOVA, or exact binomial test as appropriate. Significance was deemed when the p value was less than 0.05. For cell counts, at least 5 random fields containing myenteric plexus were counted for each analyte and each sample was performed in triplicate. Statistical analysis was performed with GraphPad Prism 5 (GraphPad Software Inc, La Jolla CA) or R (v. 2.15.1).

## Results

### Proliferative Capacity of Neural Crest Cells Ex Vivo

**Figure 1:**
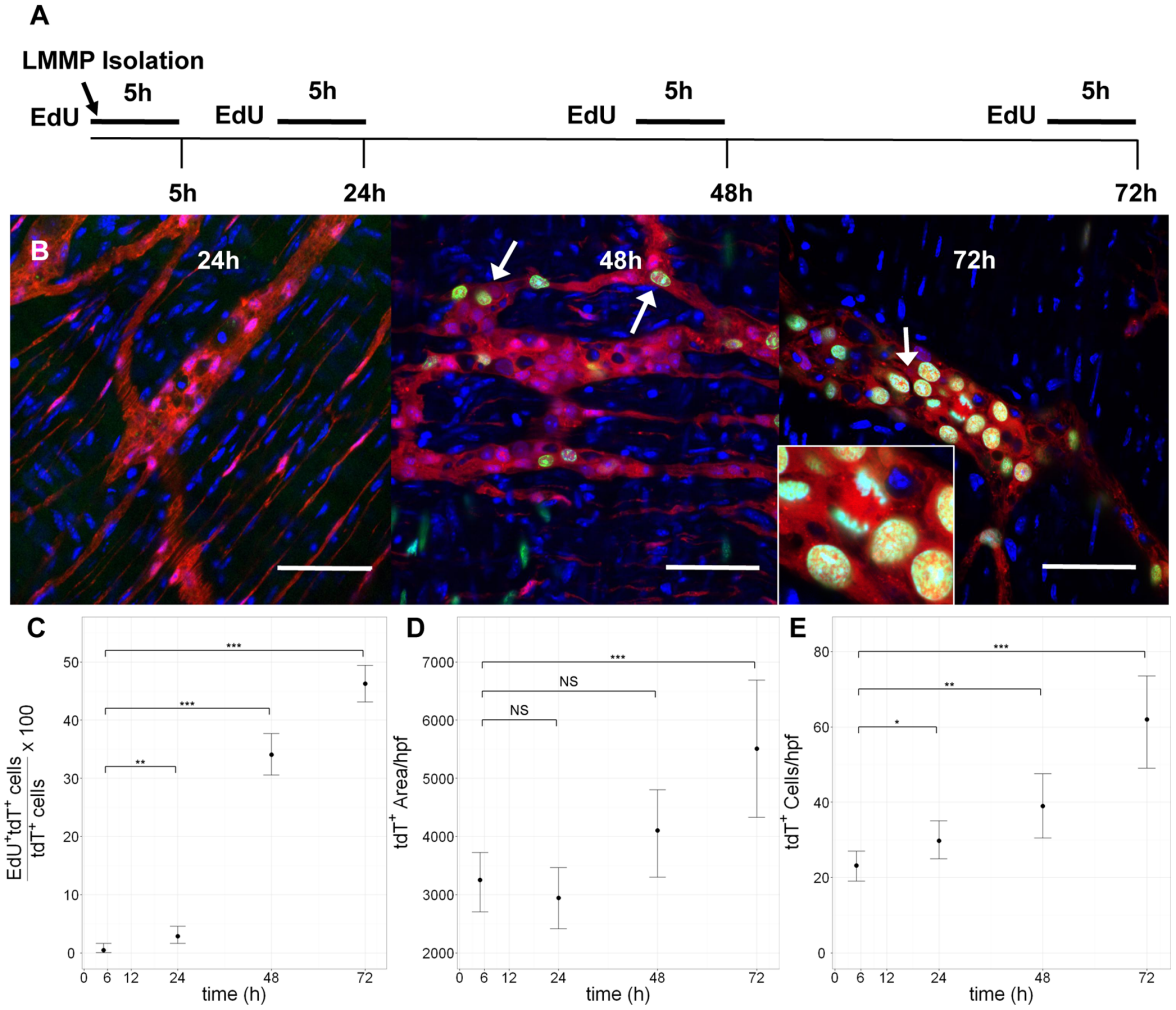
Cells of neural crest origin within myenteric plexus demonstrate EdU uptake *ex vivo*. Schematic of *ex vivo* EdU pulse experiment (A). LMMP from a *Wnt1-cre;tdTomato* mouse line was administered a 5 h “pulse” of EdU immediately after dissection (5 h) and at 24 h, 48 h and 72 h time points. While negligible at 5 h and 24 h, EdU uptake (green) was readily visible in tdT-expressing cells within ganglia (arrows) at 48 h and 72 h (B). EdU^+^ cells of neural crest origin were found in anaphase (inset) confirming that EdU uptake reflects cell division. Remarkably the rate of EdU uptake increased dramatically after 24 h reaching close to 45% of tdT-expressing cells by 72 h (C). The increase in EdU uptake after 24 h corresponded to increased total area of tdT expression per field (D) and increased total number of tdT-expressing cells per field (E) suggesting that EdU uptake reflects proliferation of cells. Area measurements (D) were made by performing automatic threshold determination on the red (tdT) channel in individual high-resolution images and measuring the area in percentage of each image with intensity above the threshold using ImageJ software. Area percentages were then converted to absolute units of µm^2^. (C) Statistical significance testing by exact binomial test; scale bar = 50 µm. (D & E) Significance by Mann-Whitney test for difference in location of non-normal populations. (C-E) NS = not significant; * = p<0.05; ** = p<0.01; *** = p<0.001; error bars indicate 95% confidence interval for the mean.

Cre recombinase under the neural crest-specific Wnt1 promoter has been used to exclusively label or genetically alter enteric neural crest cells within the mouse gut [7], [10], [38], [39]. We utilized a *Wnt1-cre;tdTomato* mouse line which allows clear visualization of cells of neural crest origin within dissected LMMP [7]. After culturing LMMP in the presence of EdU (a thymidine analog) for 48 h, we found that 78.5%(±3.1) of tdTomato (tdT) expressing cells demonstrated EdU uptake. Proliferation was largely confined to cells of neural crest origin as 74.5% (±6.6) of all EdU^+^ cells expressed tdT. In order to examine the time course at which proliferation occurs, we administered 5 h pulses of EdU immediately after LMMP dissection and at intervals up to 72 hours (Figure 1A). We detected virtually no EdU^+^ cells at the 5 h and 24 h time points. Proliferation rapidly increased at 48 h and 72 h, ultimately reaching 45% of tdT^+^ cells by 72 h (Figure 1B and C). EdU^+^ neural crest cells were frequently seen in anaphase (Figure 1B, inset) confirming that EdU uptake indicates cell division. A similar high percentage of EdU uptake in cells of neural crest origin was observed by flow cytometry (Figure S1A). Uptake corresponded to an increase in the total area of tdT expression per examined field (Figure 1D) and an increase in the total number of tdT-expressing cells per field (Figure 1E), confirming that it reflects a true expansion of neural crest cells within the tissue. Noticeably, EdU uptake first occurred primarily in cells within the ganglia (Figure 1B, arrows) suggesting this to be the location of ENSCs.

### EdU^+^ Cells Demonstrate Sphere-forming Potential

**Figure 2:**
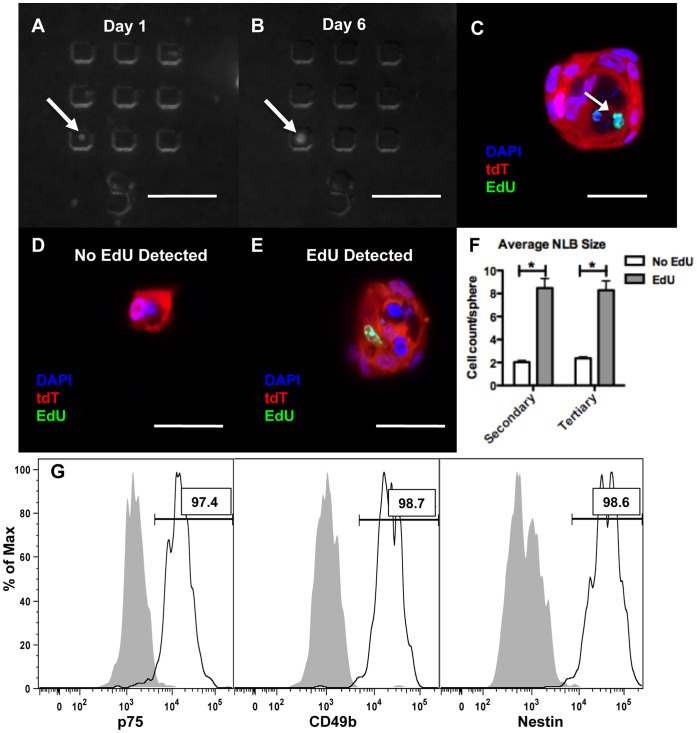
EdU^+^ cells have sphere-forming potential and express ENSC markers. Following a “pulse” with EdU, LMMP was digested and cells were cultured in media without EdU for a prolonged “chase” (up to 25 days). After either 1 or 2 passages, dissociated cells were seeded into microwells allowing tracking of single cells. Using fluorescence microscopy, single tdT-expressing cells (A, arrow) were observed that formed NLBs (B, arrow). Upon fixation and labeling of EdU, subsets of NLBs were found that contained EdU^+^ cells (C, arrow). The majority of NLBs remained small (≤3 cells/sphere cross-section) and none of these contained EdU+ cells (D). However, EdU was detected in a substantial subset of large NLBs (>3 cells/sphere cross-section) (E). The average sphere size was larger in NLBs that EdU was detected, a difference that was statistically significant for both secondary and tertiary NLBs (F). Flow cytometric analysis was performed on LMMP that was pulsed with EdU at 48 h and gated for tdT^+^/EdU^+^ single cells with debris and doublets excluded. The >97% of these cells co-expressed ENSC markers (white histogram) including p75, CD49b and Nestin (D, gray histogram is no primary control). * = p<.0001 by t-test. Error bars indicate SEM. Scale bar: 100 µm in A and B, 25 µm in C, D, and E.

The ability of single cells to form NLBs over multiple passages has been used as an assay for self-renewal and “stemness” [17], [40]. We digested LMMP that had been in culture for 48 h and pulsed with EdU for 12 hours, and cultured the dissociated cells in media without EdU for up to 25 days. Cells were seeded into microwells after 1 or 2 passages to allow clonal analysis of sphere-forming potential. Single tdT-expressing cells (Figure 2A, arrow) were found that formed both secondary and tertiary NLBs (Figure 2B, arrow) after multiple days in culture. Of the NLBs that could be traced to single cells on day 1, only a small fraction (10.1% (7/69) of secondary and 14.3% (18/126) of tertiary NLBs) became large NLBs (>3 cells/cross-section) after 6–7 days in culture. Upon EdU labeling, cells with EdU uptake could be detected in a subset of NLBs suggesting that the clonal cell from which the sphere was derived contained EdU (Figure 2C, arrow). EdU was not found in any of the secondary (0/62) or tertiary (0/108) NLBs that remained small (≤3 cells/cross-section) (Figure 2D). However, it was detected in 57.4% (4/7) of secondary and 27.8% (5/18) of tertiary NLBs that were large (>3 cells/cross-section) suggesting a correlation with EdU positivity and sphere-forming potential (Figure 2E). Overall, secondary and tertiary NLBs in which EdU was detected contained a dramatically larger number of cells per cross-section compared to their EdU negative counterparts (Figure 2F, p<.0001).

Certain types of stem cells including NSCs have been shown to partition chromosomes asymmetrically during cell division [41], [42], [43], a stem cell property known as the ‘immortal strand hypothesis’ [44]. Unlike embryonic stem cells or fibroblasts in which BrdU signal became undetectable after seven cell divisions, NSCs retained BrdU signal in 8.7% of cells after 10 days (∼10 population doublings) [42]. Similar to NSCs, we detected EdU signal in a small fraction of cells within the sphere (figure 2C and 2E). Of the tertiary NLBs in which EdU was detected, signal was found in 14.9% (±2.9) of cells. Given that these cells had been passaged twice and cultured without EdU for 25 days it suggests that asymmetric segregation of DNA during cell division is also a property of ENSCs.

### EdU^+^ Cells Express Markers of ENSCs

A characteristic of ENSCs is their expression of certain markers including Nestin, p75, and CD49b [11]. We digested LMMP following 48 h in organotypic culture, including a 12 h EdU pulse, and performed flow cytometry on single cells that were fixed, labeled for EdU, and stained for ENSC markers. Greater than 90% of tdT-expressing EdU^+^ cells also expressed p75, CD49b, Nestin (Figure 2G), and GFAP (data not shown) suggesting that they represent ENSCs. This contrasted with the non-neural crest EdU^+^ fraction (tdT^−/^EdU^+^) where the majority of cells did not express p75, Nestin or GFAP (although did express CD49b) (Figure S1B).

### EdU^+^ Cells Express Neuronal Markers

**Figure 3:**
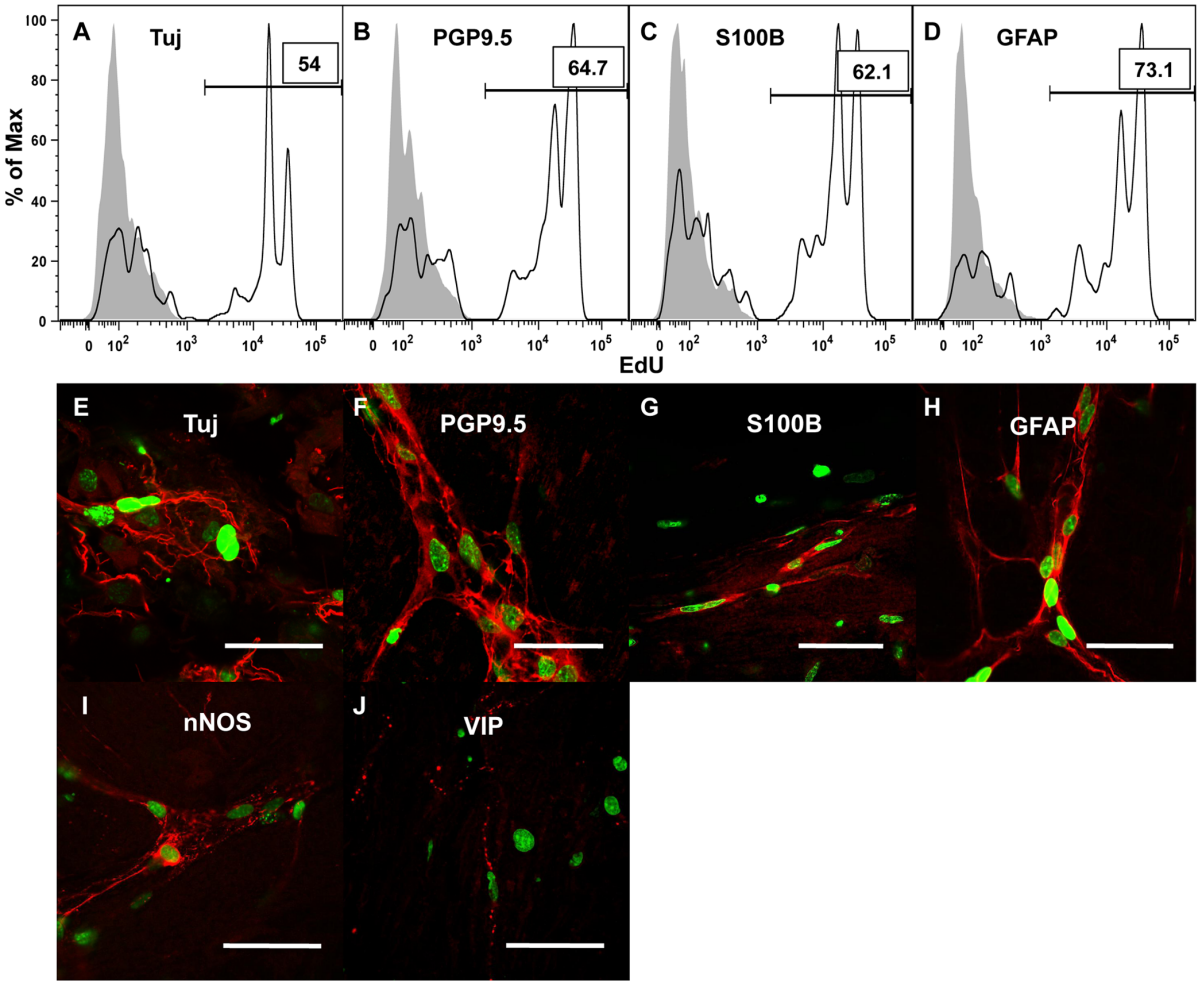
EdU uptake occurs in neurons and glia *ex vivo*. Flow cytometric analysis was performed on LMMP that was cultured with EdU for 72 h and gated for single cells that co-expressed tdT and the specified neuronal or glial marker (white histogram). EdU uptake was detected in 54% of Tuj/tdT^+^ cells (A), 64.7% of PGP9.5/tdT^+^ cells (B), 62.1% of S100ß/tdT^+^ cells (C), and 73.1% of GFAP/tdT^+^ cells. The gray histogram represents LMMP cultured with EdU for only 24 h was used as a negative control. After culturing LMMP for 48 h in media containing EdU then chasing for 5 days in differentiating media without EdU, immunofluorescence revealed cells with EdU uptake (green) that immunostained for neuronal and glial markers (red) including Tuj (E), PGP9.5 (F), S100ß (G), GFAP (H), nNOS (I), and VIP (J). Scale bars: 50 µm in E-J.

In order to evaluate for neurogenesis in our *ex vivo* system, LMMP incubated with EdU was digested after 72 hours of culture and stained for glial (GFAP and S100ß) and neuronal (PGP9.5 and Tuj) markers. By flow cytometry, EdU uptake was detected in over 50% of neural crest cells that expressed neuronal markers (54% of tdT^+^/Tuj^+^, Figure 3A white, and 64.7% of tdT^+^/PGP9.5^+^, figure 3B white). The percentage of EdU uptake was even higher for tdT^+^ cells expressing glial markers (62.1% of tdT^+^/S100ß^+^, Figure 3C white, and 73.1% of tdT^+^/GFAP^+^, Figure 3D white). To confirm neuronal EdU uptake, LMMP was cultured for 48 h with a 12 h EdU pulse, then chased in differentiating media for 5 days. Upon EdU labeling and immunostaining, EdU uptake was detected in cells that stained for pan-neuronal markers Tuj (Figure 3E) and PGP9.5 (Figure 3F), and neuronal subtypes nNOS (Figure 3I) and VIP (Figure 3J). EdU^+^ cells that stained for glial markers S100ß (Figure 3G) and GFAP (Figure 3H) were also found.

### Proliferation of Cells of Neural Crest Origin is Dependent on the PTEN/PI3K/Akt Pathway

**Figure 4:**
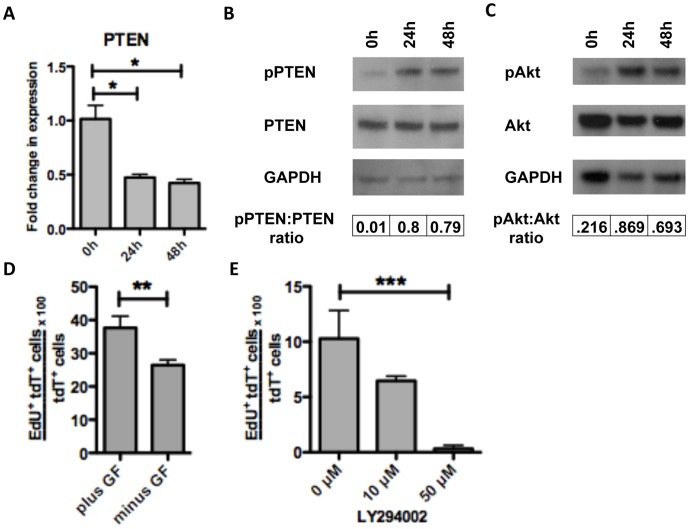
Proliferation of cells of neural crest origin is regulated by P13K/Akt signaling pathway via loss of PTEN inhibition. We observed a two-fold reduction in PTEN mRNA expression at 24 h and 28 h compared to baseline (0 h) by qRT-PCR (A). Although total PTEN protein levels did not differ there was an 80-fold elevation in the ratio of phospho-PTEN:total PTEN (where phospho-PTEN represents inactive state) at 24 h and 48 h on western blot analysis (B). This was accompanied by a 4-fold and 3-fold increase in the ratio of phospho-Akt:total Akt at 24 h and 48 h, respectively, on pixel intensity of western blot analysis (C). The presence of growth factors (bFGF, EGF, and GDNF) in media caused a modest increase in proliferation at 48 h (D). When organotypic cultures were treated with the PI3K inhibitor LY294002 (without growth factors) for 30 h, a reduction in proliferation was seen at both 10 µM and 50 µM concentrations (E). * = p<0.005 by one-way ANOVA with Bonferroni’s multiple comparison, ** = p<.05 by t-test, *** = p<.01 by one-way ANOVA with Bonferroni’s multiple comparison. Error bars indicate SEM.

We evaluated whether loss of inhibition by PTEN may account for the increase in proliferation over time. On qRT-PCR analysis, PTEN mRNA expression declined by over 50% by 24 h (Figure 4A). A similar decline in PTEN protein expression was not observed by western blot analysis (Figure 4B). One possible explanation for this discrepancy was the dramatic increase in PTEN phosphorylated on the C-terminus (Ser380) (Figure 4B), which has been associated with increased PTEN stability but decreased activity [37], [45]. Based on pixel intensity, the phospho-PTEN:total PTEN ratio was 80-fold higher at 24 h and 48 h compared with steady state levels at isolation (Figure 4B). Loss of PTEN inhibition corresponded with a rise in Akt activity. On western blot analysis, the phospho-Akt:total Akt ratio increased by over 3–4 fold at 24 h and 48 h compared with steady state at 0 h (Figure 4C). To determine the influence of growth factors (FGF, EGF and GDNF) in the media, we performed proliferation assays in the presence and absence of growth factors. While growth factors did augment proliferation, substantial EdU uptake was observed even in their absence (Figure 4D). In order to confirm that the PI3K/Akt pathway is required for proliferation, we performed a dose response experiment with the PI3K inhibitor LY294002 in the absence of growth factors. Compared with untreated control, 10 µM and 50 µM of LY294002 caused a 37% and 99% reduction in EdU uptake at 30 h (Figure 4E).

**Figure 5:**
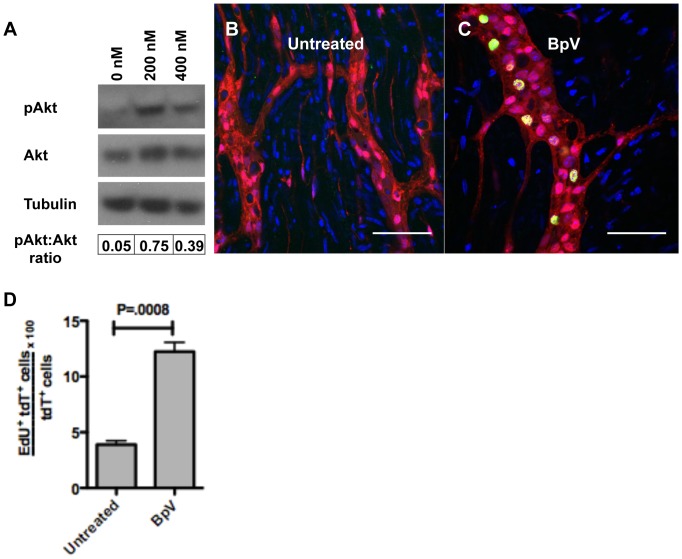
PTEN inhibition results in increased Akt activity and neural crest proliferation. Westen blot analysis of LMMP cultured in the presence of the PTEN inhibitor bpV(phen) (200 nM and 400 nM) for 7 h revealed greater than 15-fold (200 nM) and 7-fold (400 nM) increase in the ratio of phospho-Akt:total Akt based on pixel intensity (A). When organotypic cultures were treated with 200 nM of bpV(phen) (without growth factors) for 25 h inceased EdU uptake was seen in cells of neural crest origin (C) compared with untreated control (B). BpV(phen) resulted in a 3-fold increase in proliferation (D) from 3.89% (±0.37) to 12.24% (±0.83), a difference statistically significant by t-test. Scale bars: 50 µm in B and C.

Next, we assessed the effect the PTEN inhibitor bpV(phen) on proliferation. Evaluating the dose response to bpV(phen) over 7 h (also in the absence of growth factors), we found that inhibitor concentrations of 200 nM and 400 nM resulted in over 15-fold and 7-fold elevations in the ratio of phospho-Akt:total Akt respectively, relative to control (Figure 5A). Organotypic cultures incubated with EdU in the presence or absence of bpV(phen) (200 nM) for 25 h revealed that the PTEN inhibitor resulted in a 3-fold increase in proliferation compared to control (Figure 5B and C). The percent of EdU uptake increased from 3.9% (±0.37) to 12.2% (±0.83) of tdT^+^ cells with bpV(phen) (figure 5C).

## Discussion

Discrepancies between *in vivo* and *in vitro* studies have created confusion regarding the capacity for neurogenesis in the adult ENS. We and others have found that under *in vitro* conditions, ENSCs isolated and propagated from adult intestine can readily differentiate into neurons [7], [8], [9], [11], [12], [14], [16], [17], [18], [21]. However, neurogenesis has been more difficult to demonstrate *in vivo*. Neurogenesis is undetectable under steady state conditions in the adult ENS and is only seen in variable amounts under certain conditions (ie. injury, 5-HT4 agonist) [11], [13], [15], [26]. Here we introduce a novel *ex vivo* organotypic culturing system that allows us to study neurogenesis in the intact myenteric plexus over time. Unlike dissociated cells, our *ex vivo* system has the advantage of preserving the architecture of the myenteric plexus and maintaining many of the interactions within the so-called “stem cell niche”. Yet clearly it does not mimic *in vivo* conditions. In the process of preparing our *ex vivo* tissue cultures, ENSCs are removed from the influences of systemic factors supplied by the vasculature and by neural connections with the mucosal layers and extraintestinal tissue. Furthermore, the “preparation” whereby the muscle layer is stripped from the submucosa likely causes a disruption of the extracellular matrix with release of local factors. Finally, the tissue is then influenced by extrinsic factors in the culturing method including increased oxygen tension and nutrients and other factors supplied in the media. Despite these inherent differences with the *in vivo* state, our *ex vivo* system has demonstrated several important findings. First, the ENS appears to have a remarkably high capacity for proliferation with the majority of cells of neural crest origin demonstrating EdU uptake by 48 h. Next, we used our model to test the effect of compounds on promoting or delaying proliferation. We found that small molecule inhibitors of PI3K and PTEN either markedly prolonged the lag phase (PI3K inhibitor LY294002) (Figure 4E) or shortened it (PTEN inhibitor bpV(phen) (Figure 5D). Thus this system offers the potential for finding other compounds that regulate quiescence possibly through high-throughput screening. Such compounds may have therapeutic benefit in restoring bowel function following surgery or for intestinal neuromuscular disorders.

Finally, we were able to gain insight into the regulatory process that controls the transition from quiescence to proliferation. Proliferation appears to be driven by PTEN inhibition via phosphorylation of the C-terminal tail which leads to decreased phosphatase activity. The exact kinase(s) responsible for this phosphorylation are unknown but a recent publication has implicated Src and casein kinase 2 alpha [46]. Several lines of evidence support our finding that PTEN is an important regulator of ENSC quiescence. In studies using conditional PTEN deletions with *PTEN^fl/fl^* mice, PTEN appears to have a generalized role in suppressing proliferation in neural and haematopoietic stem cells [30], [31], [32], [33], [34], [35]. PTEN activity also affects proliferation and pluripotency in embryonic stem cells [29]. Furthermore, loss of PTEN in humans has been associated with intestinal ganglioneuromatosis [47], [48] and a mouse conditional knockout of PTEN in the ENS resulted in enteric ganglia hypertrophy and hyperplasia and a chronic intestinal pseudoobstruction disease phenotype [36]. Finally, local inhibition of PTEN following CNS or peripheral nerve injury has been shown to promote regeneration through neurite outgrowth [49], [50], [51]. Thus manipulation of PTEN may be a therapeutic strategy for promoting regeneration of the ENS. Transient reduction in PTEN activity is likely the best strategy since complete elimination of PTEN could cause ganglioneuromatosis or other functional disturbances, and may ultimately deplete the stem cell reserve [30], [34], [35].

Our *ex vivo* system likely reflects an injury state with disruption of the neural environment leading to suppression of PTEN. A possible explanation for the vast difference in proliferation seen our *ex vivo* system compared with *in vivo* injury models may be what the *ex vivo* system lacks. Unlike *in vivo*, it is not influenced by the inflammatory response to injury including systemic cytokines and the local influx of inflammatory cells. In the CNS, inflammation has been shown to impair neurogenesis following injury [52]. NSC function and neurogenesis was restored using indomethacin, a common non-steroidal anti-inflammatory [52]. Ultimately, future *in vivo* studies should examine whether a combined approach of inhibiting PTEN and reducing inflammation will enhance regeneration of the ENS.

## Supporting Information

Figure S1 **Majority of tdT^+^ cells are EdU^+^, and EdU^+^/tdT^−^ cells do not express p75 or Nestin.** When flow cytometry was performed on LMMP cultured with EdU for 72 h, nearly 60% of tdT-expressing cells demonstrated EdU uptake (A). Flow cytometry of LMMP pulsed with EdU at 48 h revealed that the majority of dividing non-neural crest cells (tdT^−/^EdU^+^ cells) did not express p75 or Nestin; however 63% expressed CD49b (B).
(TIF)
