# Supplementary figures and images for: Ex Vivo Neurogenesis within Enteric Ganglia Occurs in a PTEN Dependent Manner
Source: PLoS One. 2013 Mar 19;8(3):e59452. doi: 10.1371/journal.pone.0059452 (PMC3602370; doi:10.1371/journal.pone.0059452)

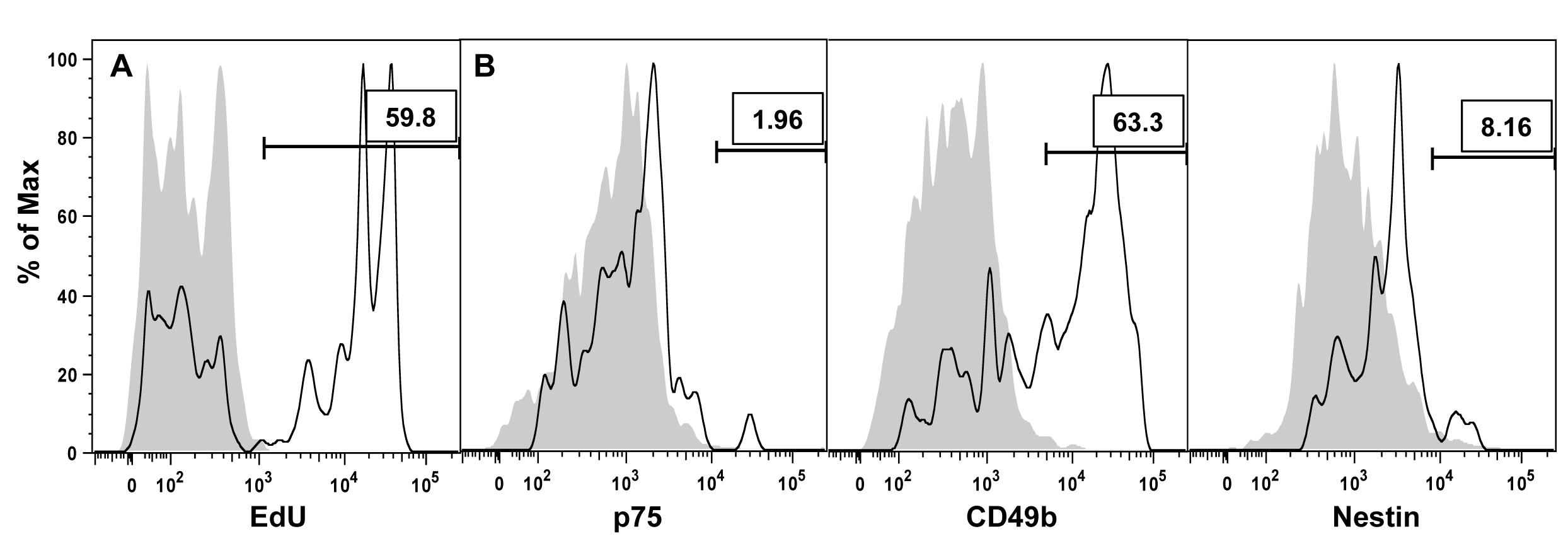

Supplement: Figure S1 — Majority of tdT+ cells are EdU+, and EdU+/tdT− cells do not express p75 or Nestin. When flow cytometry was performed on LMMP cultured with EdU for 72 h, nearly 60% of tdT-expressing cells demonstrated EdU uptake (A). Flow cytometry of LMMP pulsed with EdU at 48 h revealed that the majority of dividing non-neural crest cells (tdT−/EdU+ cells) did not express p75 or Nestin; however 63% expressed CD49b (B). (TIF) [file pone.0059452.s001.tif]
